# *De novo* large rare copy-number variations contribute to conotruncal heart disease in Chinese patients

**DOI:** 10.1038/npjgenmed.2016.33

**Published:** 2016-09-14

**Authors:** Christopher C Y Mak, Pak Cheong Chow, Anthony P Y Liu, Kelvin Y K Chan, Yoyo W Y Chu, Gary T K Mok, Gordon K C Leung, Kit San Yeung, Adolphus K T Chau, Chelsea Lowther, Stephen W Scherer, Christian R Marshall, Anne S Bassett, Brian H Y Chung

**Affiliations:** 1 Department of Paediatrics & Adolescent Medicine, LKS Faculty of Medicine, The University of Hong Kong, Hong Kong, China; 2 Department of Obstetrics and Gynecology, Queen Mary Hospital, Hong Kong, China; 3 The Clinical Genetics Research Program at The Centre for Addiction and Mental Health, The Dalglish Family 22q Clinic at The University Health Network, and The Department of Psychiatry at The University of Toronto, Toronto, ON, Canada; 4 The Centre for Applied Genomics and Program in Genetics and Genome Biology, The Hospital for Sick Children, Toronto, Ontario, Canada

## Abstract

Conotruncal heart anomalies (CTDs) are particularly prevalent congenital heart diseases (CHD) in Hong Kong. We surveyed large (>500 kb), rare (<1% frequency in controls) copy-number variations (CNVs) in Chinese patients with CTDs to identify potentially disease-causing variations. Adults who tested negative for 22q11.2 deletions were recruited from the adult CHD clinic in Hong Kong. Using a stringent calling criteria, high-confidence CNV calls were obtained, and a large control set comprising 3,987 Caucasian and 1,945 Singapore Chinese subjects was used to identify rare CNVs. Ten large rare CNVs were identified, and 3 in 108 individuals were confirmed to harbour *de novo* CNVs. All three patients were syndromic with a more complex phenotype, and each of these CNVs overlapped regions likely to be important in CHD. One was a 611 kb deletion at 17p13.3, telomeric to the Miller–Dieker syndrome (MDS) critical region, overlapping the *NXN* gene. Another was a 5 Mb deletion at 13q33.3, within a previously described critical region for CHD. A third CNV, previously unreported, was a large duplication at 2q22.3 overlapping the *ZEB2* gene. The commonly reported 1q21.1 recurrent duplication was not observed in this Chinese cohort. We provide detailed phenotypic and genotypic descriptions of large rare genic CNVs that may represent CHD loci in the East Asian population. Larger samples of Chinese origin will be required to determine whether the genome-wide distribution differs from that found in predominantly European CHD cohorts.

## Introduction

Congenital heart diseases (CHDs) are the most common birth defect and a major cause of morbidity and mortality.^1^ Conotruncal heart defects (CTDs) are CHDs affecting the cardiac outflow tract. These include tetralogy of Fallot (ToF), pulmonary atresia with VSD (PAVSD), truncus arteriosus, interrupted aortic arch, transposition of the great arteries and double outlet right ventricle. Overall, CTDs have an estimated prevalence of 11.6 per 10,000 live births.^2^ Figures available for ToF, the most common CTD, indicate a prevalence of 2.7 per 10,000 live births in Europe^2^ and 4.7 per 10,000 live births in the United States.^3^ In Taiwan, the prevalence has been reported to be 6.26 per 10,000 live births.^4^ Although Hong Kong does not have the prevalence data for CTD, in terms of the proportion of all CHD, pulmonary outflow tract obstruction alone accounts for 31.1% of all symptomatic Chinese neonates with CHD in Hong Kong, almost double the proportion reported in western studies,^5^ implying that this group of congenital abnormalities may have greater representation in Asian populations.

Although it is thought that genetic factors have an important role in CHD, only about 11% of patients receive a genetic diagnosis.^1^ In addition to chromosomal abnormalities and rare single-nucleotide variants, a growing proportion of the molecular diagnosis is attributed to rare copy-number variations (CNVs). Multiple studies show an increased burden of rare CNV in patients affected with ToF compared with controls, and several recurrent loci have been reported to be pathogenic for ToF, the most notable being the deletion responsible for 22q11.2 deletion syndrome.^6–12^ Nevertheless, Chinese studies are lacking.

Recognising the importance of CTDs in Chinese patients, and the wide spectrum of CNVs contributing to CTDs in other cohorts, our aim was to discover important CNVs in a Chinese cohort, focusing on the large (>500 kb) CNVs, readily detectable on clinical microarray platforms. We hypothesised that large rare structural variants that overlap the coding sequence of one or more genes would be associated with CTDs, over and above the recurrent 22q11.2 deletion we previously showed to be prevalent in this cohort.^8^ We present case examples characterising the lifetime phenotype of patients harbouring CNVs that are likely to be the most clinically significant.

## Results

### Patient demographics

The 116 patients (62 males, 54 females) with CTD recruited from the adult CHD clinic had a mean age±s.d. of 31.0±8.4 years that showed a normal distribution (range 17–53 years, skewness 0.74, kurtosis 0.031). All of the patients recruited with CTD had either ToF (81.9%, *n*=95) or PAVSD (18.1%, *n*=21).

### Large rare CNVs

Using stringent criteria and methods,^10^ rare CNV calls were obtained for 108 (93.2%) of the 116 subjects and therefore these subjects were used as the CNV discovery cohort. Filtering for only CNVs found in <1% of platform-matched controls, a total of 306 rare autosomal CNVs were identified in these 108 subjects. The subsequent analysis focused on CNVs that were large (defined as >500 kb), rare (<1% in all platform-matched and ethnicity-matched control groups, total=5,902), and overlapped the exons of genes. Ten large CNVs from ten patients met these criteria (Table 1). Of note, the sex distribution of these 10 patients was skewed, with 7 females and 3 males, although the overall cohort had an excess of male patients. None of the large CNVs identified overlapped the smaller (<500 kb) rare CNVs found in the sample of 108 patients. The clinical features of these 10 patients are summarised in Table 2.

### Clinical cases and candidate genes of significant large rare CNVs

Of these 10 CNVs, three were *de novo* large rare CNVs found in syndromic patients. First is a large duplication at 2q22.3 (Patient 1), the second, a 5 Mb deletion at 13q33.3 (Patient 9) and the third, a 611 kb deletion at 17p13.3 (Patient 10). Below we discuss in detail the phenotypic and molecular features of these three patients.

### 2q22.3 duplication overlapping the *ZEB2* gene

**Figure 1:**
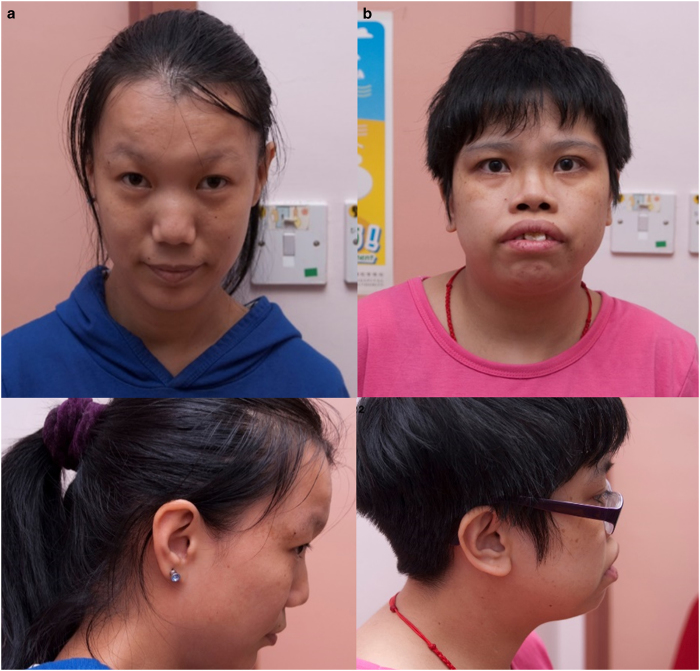
Patient facial phenotypes. (**a**) Patient 1 with 1.8 Mb gain at 2q22.3 involving the *ZEB2* gene. (**b**) Patient 10 with 611 kb loss at 17p13.3 involving the *NXN* gene. No consent for clinical photos for Patient 9.

Patient 1 (female, age 32 years) was born with ToF and patent foramen ovale. She had mild intellectual disability and adjustment disorder. She also has a history of dysfunctional uterine bleeding. On examination, she was dysmorphic, but did not fit the facial features of Mowat–Wilson syndrome. The most remarkable observation was that she had a hypernasal voice (Figure 1a).

A *de novo* 2.1 Mb copy-number gain was found at 2q22.3, which overlaps the *ZEB2* gene. Deletions of this gene are a known cause of Mowat–Wilson syndrome (OMIM#235730) in which conotruncal heart disease have been described.^13,14^ However, there have been no known reports of a duplication leading to the Mowat–Wilson syndrome phenotype. Such a large gain in copy-number may disrupt regulatory elements, such as the expression levels of *ZEB2*, which would subsequently affect transcription downstream.

The *ZEB2* gene (previously known as *ZFHX1B*, or *SIP1*) is widely expressed in human embryological development, including in the heart.^15^ The protein encoded by this gene, is SMAD-interacting protein-1 (ref. 16), which acts to activate or repress transcription by binding to regulatory sequences of E-boxes.^17^ SMAD proteins are present in the cytoplasm to mediate transforming growth factor β (TGF-β) signals from receptors of the cell-surface into the nucleus,^18^ and hence the TGF-β group of cytokines are likely to have an important role in embryological development,^18^ including cardiac outflow tract formation. Loss of *ZEB2* in the mouse is associated with variable heart defects,^19^ similar to the cardiac anomalies with deletions of this gene in human Mowat–Wilson syndrome.

### A 13q33.3 deletion in a critical region for CHD

Patient 9 (female, age 21 years) has a complex cardiac anatomy of dextrocardia, PAVSD, and patent ductus arteriosis. She was born cyanotic and small for dates, with a birth weight of 2.5 kg. Neurological assessment up to 8 months was apparently normal, however, at age 15 months, motor and speech delays were noted. She also had a brain abscess complicated by focal seizures at age 21 months. The head circumference was below the 3rd centile even before the occurrence of the brain abscess, however, and remained at the same percentile after the resolution of this infection. Subsequently, there was overall slow progress in intellectual function and the patient had attended a special school. On examination, she had mild facial dysmorphic features with low set ears and hypernasal speech (no clinical photos).

**Figure 2:**
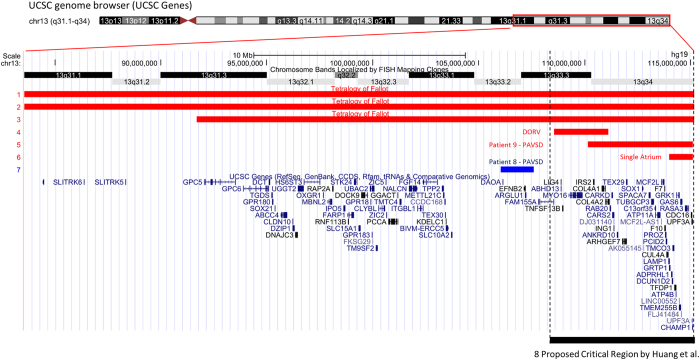
CNVs with conotruncal heart disease in the 13q31-34 region. 1–3—three cases of reported ToF with loss CNVs of approximately 20–30 Mb (Ballarati *et al.* and Quelin *et al.*).^22,23^ 4—deletion in patient with double outlet right ventricle, overlapping genes *COL4A1* and *COL4A2* (McMahon *et al.*).^25^ 5—deletion in Patient 9 from the current study with PAVSD, overlapping genes *COL4A1* and *COL4A2*. 6—deletion in patient with single atrium (Yang *et al.*).^21^ 7—duplication in Patient 8 from the current study with PAVSD. 8—CHD critical region proposed by Huang *et al.*^20^

In this patient, a large *de novo* 4.9 Mb deletion was found at 13q33.3, detectable by G-banding karyotype performed after the microarray. The CNV overlaps 41 genes, some notable among these were *TFDP1, GAS6, COL4A1, COL4A2 and SOX1* (Figure 2). Given the size, and the known critical region, this CNV was clinically classified as pathogenic.

**Figure 3:**
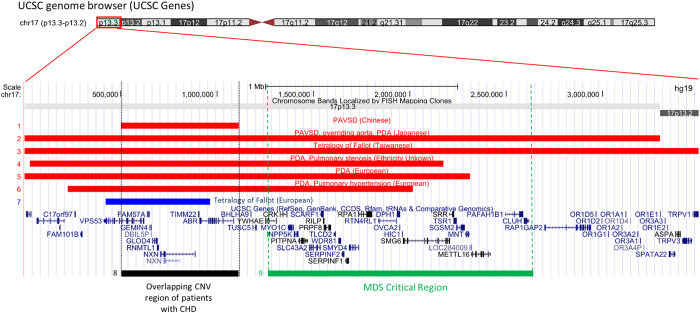
CNVs associated with congenital heart disease in the 17p13.2-13.3 region and relationship to the Miller–Dieker Syndrome (MDS) Critical Region. Deletion in Patient 10 in this study overlapping the *NXN* gene. Deletion in patient with PAVSD, overriding aorta and PDA (Kowase *et al.*).^31^ Deletion in patient with Tetralogy of Fallot (Chen *et al.*).^30^ Deletion in DECIPHER Patient 4,350 with PDA, PS. Deletion in patient with PDA (Serrenath Nagamani *et al.* 2009, patient 5). Deletion in patient with PDA, Pulm Hypertension (Shiff *et al.* 2010, patient B). Duplication in patient with ToF, CNV also overlapping *NXN* gene (Silversides *et al.*, Case 34).^10^ Overlapping region of CNV in Patient 10 with other large deletions associated with CHD, a possible critical region for CHD expression. Previously proposed critical region for MDS within the region of genes *YWHAE* and *PAFAH1B1* (Cardoso *et al.*).^50^

The 13q33.2-33.3 region has previously been proposed to be a critical region for CHD^20,21^ (Figure 2). Huang *et al.* summarised deletions with different CHD phenotypes such as ToF, ostium secundum,^22^ coarctation of the aorta,^23^ interauricular communication,^23^ pulmonary valve stenosis,^24^ patent ductus arteriosus,^24^ ASD/VSD^20^ and single atrium.^20^ Looking at conotruncal defects only, three cases of ToF have so far been reported to have very large deletions that overlap this critical region.^22,23^ McMahon *et al.* also reported a case of double outlet right ventricle within this proposed critical region with a 2.5 Mb deletion at 13q33.3-33.4 (Figure 3).^25^ They speculated that the collagen genes *COL4A1* and *COL4A2* are candidate genes for CHD as they are likely to have a role in cardiac development. The CNV found in our patient falls within the proposed critical region, and has a complex phenotype of PAVSD, dextrocardia, patent ductus arteriosus and situs solitus. It is interesting that within the small region of overlap with the CNV reported by McMahon *et al.* are the genes *COL4A1* and *COL4A2*, which agrees with their hypothesis. However, the 13q33.3 deletion of Patient 9 also overlaps a smaller 1.1 Mb deletion with single atrium reported by Yang *et al.* that did not include either of these two collagen genes.^21^ Of note, in our cohort, there was another large copy-number gain at 13q33.2-33.3 (patient 8), overlapping five genes (*EFNB2*, *ARGLU1*, *DAOA-AS1*, *DAOA* and *LINC00460*), further suggesting the potential importance of this region and ephrin-B2 signalling in CHD.^26^

Summarising all the evidence so far, we posit that deletions of 13q33.3 are strongly associated with CHD but that narrow critical regions, or candidate genes, remain to be confirmed with more cases.

### 17p13.3 deletion telomeric to the critical region of Miller–Dieker Syndrome (MDS)

Patient 10 (Female, age 27 years) is known to have PAVSD. She required multiple surgeries, including a left modified Blalock-Taussig (LmBT) shunt for left pulmonary artery stenosis at age 7 months. Subsequently, closure of VSD and right ventricular outflow tract reconstruction was performed at the age of 7 years. She is currently on warfarin due to atrial fibrillation. She had a history of epilepsy since age 17 years, initially presenting with petit mal seizures with normal EEG, controlled with sodium valproate. She studied in a special school because of mild intellectual disability. Her other medical history includes scoliosis, left hip dysplasia requiring total hip replacement, severe myopia and amblyopia, as well as primary amenorrhoea (diagnosed at age 17 years). On examination, she had dysmorphic facial features (Figure 1b).

The CNV analysis revealed a *de novo* 611 kb loss at 17p13.3 overlapping 10 genes, including *NXN* (Figure 3). The *NXN* gene is expressed in murine heart and there is evidence that loss of the *NXN* gene is associated with CHD in mice.^27^ In humans, the gene is expressed in human heart and has a haploinsufficiency prediction score of 37.4%,^28^ as well as RVIS (Residual Variation Intolerance Score)^29^ percentile of 11.77% (i.e., the top 11.77% most intolerant of human genes to variation). The role of *NXN* in the canonical Wnt/β-catenin signalling pathway through regulation of the dishevelled protein and the gene’s subsequent role in second heart field signalling, further suggest this gene’s potential involvement in cardiac development.

Notably, a similarly sized (544 kb) rare copy-number gain overlapping the *NXN* gene was reported by another group to be associated with ToF.^10^ As PAVSD may be considered to be at the more severe end of the spectrum of the cardiac phenotype in relation to ToF, the loss of one copy at this locus may implicate a more profound cardiac developmental effect for CHD than would a gain of copy. Isolated case reports indicate a link between CHD and large deletions in this region at the resolution of the chromosome band, including two from Taiwan and Japan with ToF^30^ and PAVSD,^31^ respectively, that overlap *NXN*. This suggests that a critical region for CHD may be located in the region of the *NXN* gene (Figure 3), in addition to the more proximal MDS region (Figure 3).

## Discussion

### Large rare CNVs in conotruncal heart disease

A number of studies have explored the contribution of CNVs to conotruncal heart defects, particularly ToF. Most of these studies involved samples from predominantly Caucasian populations.^6,7,9–11,32^ Although there is evidence to suggest an increased burden of large rare CNVs in ToF patients compared with the normal population,^6,10,11^ few loci have been found to be recurrently associated with the disease, implicating substantial genetic heterogeneity.^33^ The most notable recurrent CNVs responsible for ToF are 22q11.2 deletions, followed by 1q21.1 duplications.^7,10,11^ In this study of adult Chinese patients with CTDs, after excluding those with recurrent 22q11.2 deletions (15.3%), we discovered large rare CNVs in 10 of 108 (9.3%) patients; the previously reported 1q21.1 duplication involving *GJA5* gene was not found.^7,10,11^ We were able to characterise the clinical features of these 10 patients by clinical re-evaluation and determine that three were syndromic. All three of these patients carried a *de novo* CNV harbouring genes potentially linked to cardiac development that would be considered pathogenic. The other seven large genic CNVs, all very rare and in patients with few or no reported extracardiac features, would represent variants of unknown clinical significance (VUS). For the two of these seven with parental samples available the CNVs were inherited, as is often found for complex developmental conditions.^34^

### Possible ethnic specific CNV distribution in Chinese

Whist there appears to be some similarities and overlap of CNVs in our cohort compared with the literature, such as the prevalence of recurrent 22q11.2 deletions and overlap of deletion at 5q35.3 (Supplementary Information S1), the distribution of other large rare CNVs in this Chinese CTD cohort seemed to differ to some extent from the published literature on ToF in the predominantly Caucasian samples studied. Several publications reported recurrent large (over 1 Mb) rare duplications involving the *GJA5* gene.^7,10,11^ Absence of this CNV in our cohort could be due to insufficient sample size given the ~1% prevalence in European ToF cohorts.^7^ Alternatively, 1q21.1 duplications may be rarer in East Asian populations. It is known that there can be considerable stratification of recurrent rearrangements between Asian and European populations,^35^ and this may be the case for 1q21.1 duplications. Despite our study being the largest Chinese cohort of CTD reported to date, a larger sample sizes will be needed to determine whether 1q21.1 CNVs are truly rarer in individuals of Chinese origin than in those of European descent.

With respect to the rare *de novo* CNVs found, we speculate that CNVs in the greater 17p13.3 region may be an important cause of developmental disorders in the Chinese population. In general, CNVs in this region are non-recurrent and may lead to various phenotypes other than that of typical MDS. Supporting this possibility, our group has reported cases of 17p13.3 duplication (with aortic stenosis, microcephaly and dysmorphism)^36^ and triplication (with split-hand malformation)^37^ in Hong Kong. Larger studies of the Chinese population will be needed to better classify pathogenic CNVs in the 17p13.3 region, and to determine whether there are any ethnic differences in prevalence.

### Advantages and limitations

Our study was carried out using two large groups of European controls,^10^ and one of Chinese controls,^38^ to adjudicate rarity of CNVs. The array platform and stringent criteria used for CNV calling have also been validated before.^10^ Another advantage of this study is the utilisation of an adult cohort with the availability of long-term medical information. Data regarding both development and later medical complications could be obtained as a result, giving us more information on the potential phenotypic effects of these large rare CNVs over a long period of time. Performing this study on Han Chinese exclusively also meant that we could better begin to characterize the distribution of CNVs related to CHD in this ethnic group, as well as provide documentation of the clinical presentation for reference. Nevertheless, despite being the largest Chinese study so far of CNVs in CHD, our sample size was not sufficient for CNV burden analysis. Furthermore, not all parental samples were available for determination of *de novo* status of these CNVs.

## Conclusion

This study of 116 Chinese patients with conotruncal heart defects identified ten large (>500 kb) rare genic CNVs, three of which were determined to be *de novo* and pathogenic and the others of less certain clinical significance. Notably, we did not find the previously reported recurrent 1q21.1 duplication in our cohort. The small sample size precludes concluding whether or not the distribution of rare CNVs differs in the Chinese population from that observed in European populations. We also provide further evidence for the pathogenicity of the 17p13.3 and 13q33.3 loci, involving the candidate genes *COL4A1* and *COL4A2*, and *NXN*, respectively.

## Materials and methods

### Patients and samples

**Figure 4:**
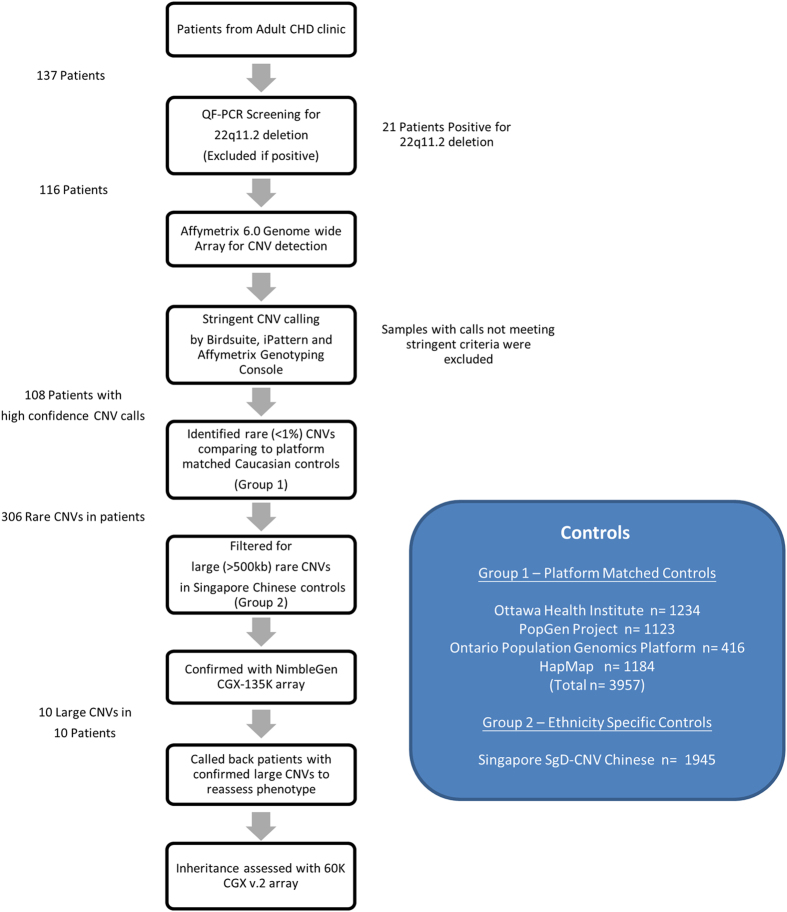
Study protocol for CNVs associated with conotruncal anomalies.

A summary of the study design is provided in Figure 4. Adult patients (>18 years of age) with CTDs, but no prior genetic diagnosis, were prospectively recruited between February 2012 to July 2013 from the adult congenital heart disease clinic at Queen Mary Hospital, the only tertiary referral centre for CHDs in Hong Kong. All patients were self-reported to be Han Chinese. On a research basis, patients were identified by the attending cardiologist and recruited if a diagnosis of congenital CTD had been recorded. The diagnoses were determined from previous cardiology clinic notes, echocardiograms and operation records based on the cardiologist’s experience. The standard care of these patients with CHD in Hong Kong does not involve any genetic testing. Using protocols approved by the local institutional review board, written informed consent was obtained for quantitative fluorescence PCR (QF-PCR), chromosomal genome-wide microarray analysis and publication of photos (Patient 1 and Patient 10).

Genomic DNA was extracted from whole blood samples. Patients were first screened for 22q11.2 deletions by QF-PCR followed by confirmatory fluorescence *in situ* hybridisation (FISH) using a standard probe.^8^ Patients tested 22q11.2 deletion positive^8^ (*n*=21 of 137, 15%) were excluded. We included only the patients with ToF or PAVSD and excluded further two cases with persistent truncus arteriosus (*n*=1) and interrupted aortic arch (*n*=1) respectively for a more homogenous discovery cohort. Samples were sent for CNV analysis at The Centre for Applied Genomics (The Hospital for Sick Children, Toronto, ON, Canada) using the Affymetrix 6.0 SNP array.

### Stringent CNV calling criteria and control groups

Using multiple CNV calling algorithms (Birdsuite,^39^ iPattern^40^ and Affymetrix Genotyping Console), a stringent criteria was used to call the CNVs,^10^ i.e., at least 10 kb in length, spanning 5 or more consecutive array probes and called by more than one algorithm. We compared CNVs identified to two different groups of population controls and defined rare CNVs as those with a population frequency of <1% using a 50% reciprocal overlap criterion. First, we compared with CNVs analysed in the same way from 3,957 platform-matched controls,^10^ which included CNVs from the Ottawa Heart Institute Coronary Heart Disease Study, *n*=1,234; German PopGen Project, *n*=1,123;^41^ the Ontario Population Genomics Platform, *n*=416;^10^ and Hapmap3 Project,^42^ *n*=1,184. As individuals of Chinese ancestry only formed a small proportion (4.8%) of these controls, as a second stage, we compared CNVs to 1,945 Chinese subjects from the Singapore SgD-CNVdatabase^38^ in an attempt to eliminate variants with a frequency >1% that were specific to the Chinese population.

### Interpretation of CNVs

Our analysis prioritised large (>500 kb) CNVs that overlapped genes since these are more likely to be of clinical significance. CNVs not observed in any of the total 5,902 controls were classified as very rare. Only autosomal CNVs were examined.

The pathogenicity of the CNVs in relation to CTD was analysed systematically using online databases. Each CNV and gene was manually interrogated for the likelihood of causing a cardiac disease phenotype. The databases used for CNV analysis included Database of Genomic Variants (DGV),^43^ Online Mendelian Inheritance in Man (OMIM), Decipher^44^ and ISCA.^45^ Each gene overlapped by the CNV was studied using evidence from PubMed, OMIM, Mouse Genome Informatics (MGI)^46^ and ZFIN^47^ to elucidate any potential relevance to congenital heart anomalies and identified as candidate genes. Genomic parameters used were from GRCh37/hg19.

### Validation of clinically relevant CNVs

CNVs classified as putatively clinically relevant were validated using NimbleGen CGX-135K oligonucleotide array (Signature Genomics, Roche NimbleGen, Madison, WI, USA) or 60K CGX v.2 oligonucleotide array (manufactured by Agilent Technologies, Santa Clara, CA, USA; PerkinElmer, Turku, Finland), as described previously^48^ and by the manufacturers. These microarray platforms were used for clinical diagnostic purposes in our institution. The former NimbleGen array has an average resolution of 140 kb, with a higher resolution (40 kb or less) in regions thought to be of significance in human development, while the latter Agilent array has an average resolution 190 kb with a higher resolution of 28 kb in the respective regions of focus. The Genoglyphix software (Signature Genomics, Spokane, WA, USA) was used to analyse and annotate the results. Only a limited number of parental samples were obtained when calling back the patients, and trio aCGH testing was offered where possible. Several large rare CNVs detected on the Affy 6.0 platform could not be validated by the NimbleGen CGX-135K platform or quantitative real-time PCR. These CNVs were either in regions of high density of segmental duplications or in close proximity to the centromere. To eliminate these false positives, all large rare CNVs with >70% segmental duplications overlap or near the centromere (e.g., 4p11) were also excluded from this study.

### Detailed phenotyping of patients with large, rare CNVs

Patients with potentially clinically relevant CNVs were called back for reassessment by a geneticist and given genetic counselling by a genetic counsellor. Lifetime medical history was reviewed and examination was performed for these patients to systematically characterize any syndromic (two out of three features of learning difficulties, global dysmorphic facial features, hypernasal voice^49^) or other extracardiac features. This encompassed birth history, medical history including thyroid function, hearing and immunodeficiency, as well as speech, learning and behavioural difficulties. A family history of CHD, intellectual disability and congenital anomalies was also obtained.

### Ethics statement

Ethical approval was obtained from the Institutional Review Board of the University of Hong Kong and Hospital Authority of Hong Kong West Cluster.

## Figures and Tables

**Table 1:** Large rare genic CNVs identified in adult Chinese patients with conotruncal defects (listed according to cytoband)

*Cytoband*	*Patient*	*Start*	*End*	*CNV Size (bp)*	*CN*	*Very rare*^a^	*Validated*	*Inheritance*	*No. of genes*	*Putative candidate genes*	*References*
2q22.3	1	144223416	146233601	2010185	Gain	Y	Yes	*De novo*	6	*ARHGAP15, GTDC1, **ZEB2***	
5q33.1	2	151540888	152162921	622034	Gain	Y	Yes	Maternal	1	*NMUR2*	
6p21.31	3	34422354	34943632	521279	Gain	Y	Yes	Maternal	7	*SPDEF, TAF11, UHRF1BP1, SNRPC, ANKS1A, PACSIN1*	
7p21.1	4	16893610	17502331	608722	Gain	Y	Yes	NA	2	*AHR, AGR3*	
7q33	5	133760551	134367872	607322	Loss	Y	Yes	NA	6	*LRGUK, AKR1B10, AKR1B15, BPGM, SLC35B4, AKR1B1*	
16q23.1	6	76070911	77087123	1016213	Gain	Y	Yes	NA	2	*CNTNAP4, MIR4719*	
12q24.32	7	127967297	128566111	598815	Gain	Y	Yes	NA	1	*FLJ37505*	
13q33.2,13q33.3	8	106040985	107581224	1540240	Gain	Y	Yes	NA	5	***EFNB2**, ARGLU1, DAOA*	Davy *et al.*^26^
13q33.3,13q34	9	110162811	115108397	4945587	Loss	Y	Yes	*De novo*	41	*LAMP1, TMCO3, GRTP1, CARS2, ATP11A, **SOX1**, ANKRD10, CDC16, ARHGEF7, GRK1AS1, ADPRHL1, DCUN1D2, **COL4A2, COL4A1**, FAM70B, F10, ING1, RASA3, RAB20, SPACA7, CHAMP1, PROZ, ATP4B, F7, PCID2, MCF2L, IRS2, CUL4A, UPF3A, **TFDP1**, TUBGCP3, CARKD, **GAS6***	Huang *et al.*^20^, McMahon *et al.*^25^, Yang *et al.*^21^
17p13.3	10	500746	1112604	611859	Loss	<0.1%	Yes	*De novo*	10	*RNMTL1, FAM57A, GLOD4, GEMIN4, DBIL5P, ABR, VPS53, **NXN**, MIR3183, TIMM22*	Silversides *et al.*^10^

Abbreviations: CN, copy-number change; CNV, copy-number variants; NA, not available; Y, not found at all in any of the 3,957 platform control.

For all available parental samples, inheritance of CNV was determined and shown.

Candidate genes with potential importance in cardiac development are highlighted in bold font.

All genomic coordinates are provided using genome build hg19.

a Rarity for Group 1 controls (All CNVs are found in <1% of group 2—Chinese controls) indicating very rare CNVs.

**Table 2:** Clinical features of conotruncal anomaly patients with large (>500kb) rare genic CNVs

*Patient*	*CNV*	*Size (kb)*	*Sex*	*Age*	*Type of CHD*	*Dysmorphic*	*Hypernasality*	*Neuropsychiatric manifestations*	*Growth and development*	*Other medical issues*
1	2q22.3 gain	1,846	F	32	TOF, patent foramen ovale	Dysmorphic	Hypernasal speech	Adjustment disorder	Normal	—
2	5q33.1 gain	622	M	23	TOF	No		Normal	Normal	—
3	6p21.31 gain	521	M	22	TOF	No		Normal	Normal	—
4	7p21.1 gain	609	F	32	TOF	No		Normal	Normal	—
5	7q33 loss	607	M	25	TOF	No		Normal	Normal	—
6	16q23.1 gain	1,016	F	25	TOF	No		Mild intellectual disability	Precocious puberty	—
7	12q24.32 gain	599	F	22	TOF	No		Normal	Normal	—
8	13q33.2-13q33.3 gain	1,540	F	27	PAVSD	No		Normal	Secondary amenorrhoea	Eczema
9	13q33.3-13q34 loss	495	F	21	PAVSD, dextrocardia, situs solitus	Dysmorphic	Hypernasal speech	Mild intellectual disability, poor performance in mathematics	Mild motor delay	Brain abscess, VUR with urinary incontinence, bilateral genu valgum and mild external tibial torsion
10	17p13.3 loss	612	F	27	PAVSD	Dysmorphic		Mild intellectual disability, epilepsy	Global developmental delay	Left Hip dysplasia, Severe myopia and amblyopia, scoliosis, primary amenorrhoea

Abbreviations: CHD, congenital heart diseases; CNVs, copy-number variations; F, female; M, male; PAVSD, Pulmonary atresia with VSD; TOF, tetralogy of Fallot; VSD, ventricular septal defect.
